# Lipid Disturbances in Breast Cancer Patients during Chemotherapy

**DOI:** 10.3390/nursrep13040126

**Published:** 2023-10-25

**Authors:** Aikaterini Alimperti, Victoria Alikari, Maria Tsironi, Andrea Paola Rojas Gil, Dimitrios Papageorgiou, Petros Kolovos, Aspasia Panagiotou, George I. Panoutsopoulos, Maria Lavdaniti, Sofia Zyga

**Affiliations:** 1 Department of Nursing, University of Peloponnese, 22131 Tripolis, Greece; alikatrin@hotmail.com (A.A.); tsironi@uop.gr (M.T.); arojas@uop.gr (A.P.R.G.); dpapageorg@uop.gr (D.P.); pkolovos@uop.gr (P.K.); aspasi@uop.gr (A.P.); zygas@uop.gr (S.Z.); 2 Department of Nursing, University of West Attica, 12243 Egaleo, Greece; 3 Department of Nutrition Science and Dietetics, University of Peloponnese, 22131 Tripolis, Greece; gpanouts@uop.gr; 4 Department of Nursing, International Hellenic University, 57001 Nea Moudania, Greece; marlabda@nurse.teithe.gr

**Keywords:** breast cancer, chemotherapy, lipids, triglycerides, HDL, LDL, cholesterol

## Abstract

Breast cancer is the most common cancer in women. Cardiovascular diseases are common complications after chemotherapy due to the effect of the drug on lipid levels. This study aimed to explore the changes in lipid profiles in patients with breast cancer under chemotherapy. Methods: In this prospective study, 50 patients with breast cancer participated. Three biochemical–lipid hematological tests were performed: total cholesterol (TC), triglycerides (TGs), High-Density Lipoprotein (HDL-C), and Low-Density Lipoprotein (LDL-C) before initiation (pre-chemotherapy), at the start (first follow-up), and at the completion (second follow-up) of the first cycle of chemotherapy. Statistical significance was set at *p* < 0.05. Analyses were conducted using SPSS Statistical Software (version 22.0). Results: Mean TC values increased significantly at second follow-up. TGs values decreased significantly from first to second follow-up. HDL-C was significantly lower at first follow-up compared with pre-chemotherapy and was similar to the pre-chemotherapy levels at second follow-up. LDL-C values were significantly higher at second follow-up compared with pre-chemotherapy measurement. Significantly positive correlations of BMI with pre-chemotherapy LDL-C, first follow-up TC, first follow-up LDL-C, second follow-up TC, and second follow-up LDL-C were found. Conclusions: There is a statistically significant increase in the levels of TC and LDL-C in breast cancer patients during chemotherapy. This study was not registered.

## 1. Introduction

Breast cancer is the second leading contributor to cancer-related mortality after lung cancer (23%), accounting for 15% of cancer deaths [1]. An estimated 2.3 million new cases are expected to be diagnosed each year. Lung cancer accounts for 11.8% of all new cases, colorectal cancer accounts for 12.9%, prostate cancer for 11.7%, and gastric cancer for 5.6%. As with other diseases, it is caused by a complex interaction between genetic and environmental factors [2]. Until now, a few rare mutations in genes, such as Breast Cancer 1 and Breast Cancer 2, which significantly increase the risk of the disease, have been discovered, as well as many other much more common genes, each of which individually contributes to an increased risk of developing the disease [3].

Several studies have shown that malignancies and especially their worsening are related to disturbances in the levels of lipids and lipoproteins [4,5]. Metabolic syndrome and accompanying lipid disorders are epidemiologically linked to many neoplasms (hepatocellular carcinoma, colorectal, endometrial, prostate), either due to their effect on hormonal changes or because they are associated with common determinants (diet or inflammation) [6]. The lipid profile and its disturbances in patients with early-stage cancer are not only related to the disease but also to predisposing risk factors that affect lipids, such as polycystic ovary disease (for endometrial cancer) and obesity (for breast cancer) [7]. Researchers emphasize that low cholesterol levels are an important indicator of cancerous conditions but not necessarily a causative factor. Therefore, this finding usually concerns rapidly evolving malignancies, raising the suspicion of pre-existing preclinical malignancy [8]. In prostate malignancies, the results are inconsistent [9] since studies found weak or no correlation between cancer and high blood cholesterol levels [9], while lung cancer patients with lower High-Density Lipoprotein Cholesterol (HDL-C) levels had an increased risk of lung cancer development [10]. Regarding the association of breast cancer with the levels of lipids, data are controversial [11]. A recent study [12] found a positive correlation of breast cancer rates with elevated lipid levels while an earlier study [13] showed lower cholesterol levels in breast cancer patients compared to a group of healthy individuals. In a study [14] conducted in 1991–1996 involving 17,035 women, the risk of breast cancer was inversely related to the concentration of Apo-B, the primary protein responsible for Very Low-Density Lipoprotein (VLDL) and Low-Density Lipoprotein Cholesterol (LDL-C).

Chemotherapy plays a vital role in the management of breast cancer patients, serving as an indispensable treatment for both disease control and the overall survival of patients. Nevertheless, as new therapeutic approaches are introduced and life expectancy continues to increase, it is essential to acknowledge the long-term and delayed consequences of cancer treatment [15]. In particular, the employment of chemotherapy has been linked to enduring adverse effects, notably cardiovascular ailments. Cardiovascular diseases, including heart failure, myocardial ischemia, and hypertension, represent prevalent complications following chemotherapy administration [16]. Li et al. [17] conducted an analysis of 1054 breast cancer patients and 2483 healthy women (2483) stratified by age. The lipid profile status of the patients was characterized at the beginning of the diagnosis period and during chemotherapy. The lipid profiles become more deteriorated after chemotherapy, resulting in an increase in total cholesterol (TC), triglycerides (TGs), LDL-C, and apo-B concentrations. Levels of HDL-C decreased compared with their pre-chemo status [17].

Antineoplastic drug cardiotoxicity is dependent on a variety of variables, either intrinsic to the drug or intrinsic to the individual health of the patient. Regarding the drug itself, dosage, route of administration, cumulative dose, and number of chemotherapy sessions all play a role. Furthermore, the combination of the drug with other chemotherapy drugs may or may not contribute to the development of cardiac dysfunction [18]. These complications arise not only due to the direct cardiotoxicity of chemotherapy drugs but also due to their impact on serum lipid levels [19]. Several chemotherapy drugs can cause significant dyslipidemia in breast cancer patients after chemotherapy. Anthracyclines, such as doxorubicin, decrease ABCA1 gene and apoA1 expression in HepG2 cells, and ABCA1 expression in hepatocytes significantly contributes to HDL-C levels [13]. In addition, anthracyclines have been linked to a dose-related risk for cardiomyopathy and heart failure. In particular, when no risk factors are present, the tolerability of doxorubicin up to a dose of 300 mg/m^2^ is not significantly affected and the rate of heart failure is less than 2%. On the other hand, retrospective studies have indicated that an estimated 3–5% of patients with no other risk factors would develop heart failure at a dose of 400 mg/m^2^, and this percentage could even increase to 48% for a dose of >550 mg/m^2^ [18]. Taxanes are chemotherapeutic drugs that act as antimitotic agents, stabilizing microtubules in a mitotic spindle to inhibit cell cycle progression. However, substantial toxicities limit the efficacy of taxane-administered treatment regimens. It is reported that 3 to 20% of patients experience cardiotoxic events (QT interval extension, bradycardia, and atrial fibrillation) following the administration of taxanes [20]. Also, taxanes—specifically, paclitaxel treatment—significantly reduce HDL-C levels and increase hydroperoxide levels compared with those in breast cancer patients who do not receive chemotherapy [21].

Risk factors associated with patient-related drug cardiotoxicity include age (<4 years and old age), ethnicity (black race), obesity, smoking, pre-existing cardiovascular disease, cardiotoxic drug use, arterial hypertension, and diabetes mellitus, as well as metabolic disorders [22].

Chemotherapy itself can also cause endothelial dysfunction increasing insulin resistance. This leads to a decrease in cytokines, increasing the patients’ lipid profile [23]. Triglycerides (TGs) are a sensitive measure to explore the impact of adjuvant chemotherapy in patients with breast cancer. High TGs levels are linked to an increased risk of cardiovascular complications in patients with breast cancer, making them significant predictors of coronary heart disease [24]. Additionally, it has been observed that patients with breast cancer and high levels of High-Density Lipoprotein Cholesterol (HDL-C) are more likely to develop coronary heart disease [25].

Another element that should be emphasized at this point is the lipid status in patients with metastases. Breast cancer patients with metastases were found to have lower levels of TC and LDL-C than non-metastatic breast cancer patients. Hypocholesterolemia in breast cancer patients has been attributed to disease progression since the neoplastic cells consume a significantly greater amount of cholesterol, resulting in lower LDL-C levels [26,27]. Indeed, the correlation between the risk of cancer recurrence and body mass index (ΒΜΙ) is remarkable. Authors conducted a study [28] to investigate the association between body mass index (BMI) and the risk of distant metastasis and death due to breast cancer in early-stage breast cancer patients. The study included patients with positive estrogen receptors, negative estrogen receptors, and unknown tumors. The results showed that patients with a BMI ≥ 30 kg/m^2^ had a 42–46% increased risk of developing distant metastasis after 10 years compared with patients with a BMI < 25 kg/m^2^. Furthermore, patients with a BMI ≥ 30 kg/m^2^ had a 38% increased risk of death due to breast cancer after 30 years. A distinct investigation [29] conducted on patients with triple-negative (not having any estrogen or progesterone receptors or human epidermal growth factor receptor 2) breast cancer did not provide conclusive evidence of a correlation between obesity and mortality. In contrast, women with positive estrogen receptors exhibited a threefold higher risk of death.

Understanding the potential adverse consequences of chemotherapy on serum lipid levels is of utmost importance in order to enhance the well-being of individuals diagnosed with breast cancer in the forthcoming years. This includes ameliorating their chances of survival, prognosis, and minimizing the occurrence of complications. Based on the above, the aim of this study was to explore the status and the changes of serum lipids among patients with breast cancer during chemotherapy.

## 2. Materials and Methods

This is a prospective study carried out with the participation of women hospitalized in an oncology clinic of a tertiary hospital in Athens (convenience sample). Women with an independent stage of breast cancer with normal values of blood lipids participated in this study. Inclusion criteria were women aged >18 years with diagnosis of primary breast cancer of any stage who had undergone chemotherapy after total or partial mastectomy. The chemotherapy drugs used in these women were either Doxorubicin–Cyclophosphamide for six cycles every three weeks or Docetaxel for eight cycles every three weeks. Exclusion criteria were women diagnosed with metastases, history of other cancers, and presence of any cognitive or psychiatric difficulties. Women who had undergone or were currently undergoing any other therapeutic intervention other than chemotherapy (radiotherapy, hormone therapy, etc.) were also excluded. In total, 70 evaluable patients were invited to participate in the study. Finally, 50 patients agreed to take part (response rate 71%).

When the patients entered the hospital, their age and body mass index (BMI) were recorded. This record was noted because increasing age and body weight are associated with insulin resistance, which in turn affects lipid levels. The study was conducted in three phases: The first phase took place before the initiation of the first cycle of chemotherapy. The second phase took place at the start of the aforementioned treatment. The third phase took place at the completion of the first cycle of chemotherapy. In all three phases, a blood sample was obtained for biochemical control of lipids. The chemotherapy regimen consisted of six chemotherapy cycles. To perform the biochemical hematological control of lipids, the participants were informed that they must remain fasting and not smoke for 12 h before the control. The study was conducted from March 2018 until August 2020.

To conduct the study, licenses were obtained by the Scientific Committee of the Hospital (approval number 31/23.02.2018). Throughout the study, the principles of anonymity, voluntary patient participation, and data confidentiality were respected. Each participant was provided with information regarding the purposes of the study, the voluntary nature of their participation in it, as well as their right to discontinue participation and withdraw at any time. The procedures respected the ethical principles of the Declaration of Helsinki.

The quantitative variables were described using mean and standard deviations. The qualitative variables were described in terms of absolute (N) and relative (%) frequencies. ANOVA with repeated measurements was utilized to examine any variations in the measurements over time. To detect type I error caused by multiple comparisons, a significance level (μ = 0.05/Kc) was applied to the analysis based on the Bonferroni correction (K = number of comparisons). The percentages of individuals who had abnormal results in any of the three measurements were compared using the McNemar test. Pearson’s correlation coefficient (r) was used to correlate BMI with lipid levels. Statistical significance was set at 0.05, and significance values are two-sided. Statistical analysis of the data was performed using the statistical program SPSS 22.0.

## 3. Results

The mean age of the sample was 60.3 years (±11.4), the mean BMI value of women was 29.6 kg/m^2^ (±5.4), 46.0% (*n* = 23) of the participants were overweight, 38.0% (*n* = 19) were obese, and 16% (*n* = 8) were of normal weight. The clinical characteristics (tumor type, stage of tumor, medication, and cycles of chemotherapy) of participants are presented in Table 1.

Total Cholesterol (TC) values increased significantly from the first measurement (mean: 214.9 mg/dL, SD ± 35.6) to the second follow-up (mean: 218.2 mg/dL (SD ± 40.6) (*p* = 0.015). Pre-chemotherapy TGs values (mean: 139 mg/dL SD ± 57.1) were similar to the values of the first (mean: 151.7 mg/dL, SD ± 51.8) and second (mean: 123 mg/dL, SD ± 56.1) follow-ups. However, the second follow-up TGs values were statistically significantly lower than the first follow-up values (*p* = 0.003).

A statistically significant decrease in HDL-C levels was observed at the first follow-up (mean: 52.7 mg/dL, SD ± 14.4) compared with the pre-chemotherapy measurement (mean: 57.1 mg/dL, SD ± 15.5) (*p* = 0.05), while at the second follow-up, it was increased significantly (mean: 56.9 mg/dL, SD ± 13) compared with the first follow-up (*p* = 0.026), reaching similar baseline levels.

Pre-chemotherapy LDL-C levels (mean: 130.1 mg/dL, SD ± 34) were similar to levels at the first follow-up (mean: 134.8 mg/dL, SD ± 41.1). However, the values at the second follow-up (mean: 146.1 mg/dL, SD ± 57.1) were significantly higher compared with the baseline values (*p* = 0.035). The changes in participants’ lipid values during the three stages are given in the table below (Table 2).

The percentages of participants with normal and abnormal lipid values during the three stages of the study are given in the table below (Table 3). The percentages of participants with abnormal lipid values were similar throughout the follow-up period.

Statistically positive correlations were found between BMI Index and pre-chemotherapy LDL-C, first follow-up TC, first follow-up LDL-C, second follow-up TC, and second follow-up LDL-C. Correlations are shown in Table 4.

## 4. Discussion

The purpose of this prospective study was to look at serum lipids during the first cycle of chemotherapy in breast cancer patients. This study is significant because cancer, especially its progression, is related to disturbances in lipid levels [30].

This study showed that during chemotherapy in women with breast cancer, a significant increase in the levels of TC, LDL-C, and TGs disturbance occurs, while HDL-C remained at the same levels at the end of chemotherapy. Several studies [17,31] highlight the importance of monitoring lipid levels during chemotherapy in women with breast cancer.

In the present study, TC values were increased significantly from the baseline to the last chemotherapy of the first regimen. Furthermore, pre-chemotherapy TGs values were similar to values at baseline and the last chemotherapy of the first regimen. However, TGs values at the last chemotherapy of the first regimen were significantly lower than the values at the start of chemotherapy.

In the current study, HDL-C decreased significantly at the first follow-up compared with the pre-chemotherapy measurement, while at the second follow-up, it increased significantly compared with the first follow-up of chemotherapy, reaching similar baseline levels. Also, pre-chemotherapy LDL-C levels and first follow-up levels were similar. However, the values at the second follow-up were significantly higher compared with the baseline values. These results are in contrast to those of a retrospective study in China among 141 invasive breast cancer patients in which no significant difference was found between TC, TGs, and LDL-C before and after chemotherapy [32]. The different results are probably because the measurement of the lipid profile in the above study was performed after six cycles of chemotherapy and not immediately after the first chemotherapy. Another retrospective study conducted in China in breast cancer patients undergoing various chemotherapy regimens found significantly increased levels of TGs, TC, and LDL-C [33]. Li et al. (2018) [28] also revealed increased post-chemotherapy TGs, TC, and LDL-C levels but lower HDL-C levels [17]. In the study of Xu et al. (2020) [31], cholesterol, LDL-C, and TGs levels were found to be significantly elevated at the end of one month after completion of chemotherapy, while 12 months after completion of chemotherapy, only TGs and LDL-C levels continued to be at non-normal levels. The rate of dyslipidemia in breast cancer patients increased from 41.5% at the start of chemotherapy to 54.1% at 12 months after the end of chemotherapy [31]. In contrast, a meta-analysis [34] found no significant difference between total cholesterol and LDL-C levels before and after chemotherapy in breast cancer patients [32]. Qu et al. (2020) [35] also reported lower levels of TGs, TC, HDL-C, and LDL-C among female patients with breast cancer compared to healthy women. After neoadjuvant chemotherapy, TGs and LDL-C levels increased significantly, while HDL-C levels decreased significantly [35]. Changes in the lipid profile induced by chemotherapy may have implications for the patient’s response to chemotherapy.

Regarding the correlations between BMI Index and lipids, this study revealed statistically positive correlations of BMI Index with pre-chemotherapy LDL-C, first follow-up TC, first follow-up LDL-C, second follow-up TC, and second follow-up LDL-C. This positive correlation was also highlighted in the study of Okekunle et al. (2022) [36] among premenopausal women. He et al. [33] investigated the risk factors associated with elevated levels of TG, TC, and LDL-C as well as low levels of HDL-C in patients with normal lipid profiles prior to chemotherapy. After univariate and multivariate analyses, they found that BMI > 24 was an independent predictor of high TC, TG, LDL-C, and low HDL-C levels after chemotherapy.

The correlation between these variables is a less explored area and the mechanism behind this correlation is still unclear. It is hypothesized that obesity may alter drug metabolism and pharmacokinetics, leading to reduced efficacy and increased toxicity of chemotherapy [37,38]. The relationship between BMI, lipids, and chemotherapy outcomes varies depending on the specific chemotherapy regimen used, the stage of breast cancer, and individualized factors related to the patient’s clinical characteristics [21].

To facilitate the continuous evaluation of metabolic profiles during the diagnosis and treatment of breast cancer, interdisciplinary cooperation should be enhanced. Nurses are an integral part of the multidisciplinary team and play a prominent role in cancer screening and diagnosis, monitoring for potential chemotherapy-related disorders such as lipid disorders [39]. Early detection of lipid disorders and quick referrals by nurses to their team members lead to improved therapeutic outcomes in terms of reducing cardiovascular risk and the possibility of metastases. Health education, nutritional counseling stemming from evidence-based nursing and provided by nurses, as well as placing patients in the context of the therapeutic alliance help patients understand the necessity of cooperation in treatment. Therefore, high-quality nursing care favors the improvement of clinical outcomes of chemotherapy [40].

One of the limitations of this study is the non-stratification of patients into premenopausal and postmenopausal, as the levels of sex hormones affect lipid metabolism. Also, the type of chemotherapy the patients received was not recorded as the chemotherapeutic agent affects the patients’ serum lipid levels. Another limitation of the study is that insulin resistance was not studied. Insulin resistance is a risk factor for developing breast cancer, possibly because estrogen or Insulin-Like Growth Factor-I (IGF-I) levels are elevated. Insulin resistance has been associated with obesity, dyslipidemia, arterial hypertension, and impaired glucose tolerance. Additionally, the study sample does not allow generalization of the results. Therefore, a multicenter, randomized, controlled trial should be performed to confirm the results and generate additional findings.

## 5. Conclusions

Women with breast cancer had a statistically significant increase in TC and LDL-C levels during the first cycle of chemotherapy, and a lipid disorder was also revealed. BMI was positively correlated with TC and LDL-C levels. Hence, it is imperative to carry out lipid surveillance and engage in the management of dyslipidemia as a preventive measure during the primary diagnosis stage and also throughout the course of chemotherapy in individuals affected by breast cancer. Additionally, it is crucial to conduct further investigations to determine the temporal nature of the alterations in lipid profiles and ascertain their persistent nature, thus establishing their clinical significance for patients. Finally, given that the current investigation solely examined a restricted number of lipid alterations in reaction to chemotherapy, it would be advantageous to explore a broader spectrum of changes in the lipid composition of females diagnosed with breast cancer who are receiving chemotherapy. Additionally, it would be of value to assess the impact of distinct categories of chemotherapy medications on lipid concentrations.

## Figures and Tables

**Table 1:** Clinical characteristics of patients regarding the type of tumor, stage of tumor, medication, and cycles of chemotherapy.

	Stage of Tumor	Medication	Cycles of Chemotherapy
Type of tumor: Invasive breast carcinoma of no special type (NST)/infiltrating duct carcinoma NOS			
1.	T1cN2a	AC-TXT/3 weeks	6
2.	T2pN2aMx	AC-TXT/3 weeks	8
3.	T2N0	TXT-Pertuzumab-Trastuzumab/3 weeks	6
4.	T1(2)N0	TXT/3 weeks	3
5.	T3mNx	AC/3 weeks	6
6.	T1cN0	AC-TXT/2 weeks	8
7.	T2(2)N2a	AC-TXT/3 weeks	8
8.	T1	AC-TXT/2 weeks	8
9.	T4	FEC-TXT/3 weeks	6
10.	T2(m)N3a	AC-TXT/3 weeks	8
11.	T1bN1mi(cn)	CNF/3 weeks	6
12.	T2N3	Eribulin Mesilate/2 weeks	6
13.	T1cN0	AC-TXT/3 weeks	8
14.	T4	AC-TXT/3 weeks	6
15.	T4N1	TXT/3 weeks	8
16.	T3N3a	AC-TXT/3 weeks	8
17.	T2N1	AC/3 weeks	8
18.	T4	Carboplatin and Gemcitabine/3 weeks	6
19.	T2N3a	AC/3 weeks	6
20.	T2N1a	AC-TXT/3 weeks	8
21.	T2N0	AC-TXT/3 weeks	6
22.	T1cNx	AC-TXT/3 weeks	6
23.	T2	FEC/3 weeks	6
24.	T2	AC/3 weeks	6
25.	T1cN1mi	AC/2 weeks	6
26.	T2N1	Eribulin Mesilate/1 weeks	6
27.	T2N1a	AC-TXT/3 weeks	8
28.	T2N1c	AC/3 weeks	6
29.	T2NO	Epirubicin-AC/3 weeks	6
30.	T(m)1cN2a	AC/2 weeks	6
31.	T1bN1mi	AC-TXT/3 weeks	6
32.	T1cNx	AC/3 weeks	6
33.	T1N0	Cisplatin-TXT/3 weeks	8
34.	T2N1	Cisplatin-TXT/3 weeks	6
35.	T2N0	AC - TXT/3 weeks	8
36.	T2	FEC/3 weeks	6
37.	T2N0	AC-TXT/2 weeks	8
38.	T2	AC-TXT/3 weeks	8
39.	T1N2M0	AC/3 weeks	4
40.	T2	AC/3 weeks	6
41.	T2N1a	AC-TXT/2 weeks	8
42.	T1cN3aMx	AC-TXT/3 weeks	8
43.	T2	Fulvestrant -/1 week	6
44.	T3N1	AC-TXT/3 weeks	8
45.	T1cN2a	AC/3 weeks	6
46.	T2N0	AC-TXT/2 weeks	8
47.	T1	AC/3 weeks	6
Type of tumor: Invasive lobular carcinoma, classical subtype			
48.	T2Na	AC-TXT/2 weeks	8
49.	T(m)3N2a	AC-TXT/2 weeks	8
50.	T4	AC/3 weeks	8
Age (years) (Mean ± SD)			60.3 (±11.4)
BMI kg/m^2^ (Mean ± SD)			29.6 (±5.4)

NOS: Not otherwise specified; NST: No Specific Type; TXT: Docetaxel; AC: Adriamycin and Cyclophosphamide; FEC: 5-fluorouracil, Epidoxorubicin, and Cyclophosphamide; CNF: Cyclophosphamide, Novantrone, and 5-Fluorouracil.

**Table 2:** The changes in lipid values during the three stages of the study (N = 50).

				*p*-Value		
	Pre- Chemotherapy	First Follow-Up	Second Follow-Up	Pre-Chemotherapy vs. First Follow-Up	Pre-Chemotherapy vs. Second Follow-Up	First Follow-Up vs. Second Follow-Up
	Mean (SD) ^1^	Mean (SD) ^1^	Mean (SD) ^1^			
TC (mg/dL)	214.9 (35.6)	218.2 (40.6)	232.2 (51.7)	>0.999	0.015	0.085
TGs (mg/dL)	139 (57.1)	151.7 (51.8)	123 (56.1)	0.392	0.368	0.003
HDL-C (mg/dL)	57.1 (15.5)	52.7 (14.4)	56.9 (13)	0.005	>0.999	0.026
LDL-C (mg/dL)	130.1 (34)	134.8 (41.1)	146.1 (57.1)	0.743	0.035	0.127

^1^ SD: Standard Deviation.

**Table 3:** The percentages of participants with normal and abnormal lipid values during the three stages of the study.

					*p*-Value		
	Values	Pre-Chemotherapy Ν (%)	First Follow-Up Ν (%)	Second Follow-Up Ν (%)	Pre- Chemotherapy vs. First Follow-Up	Pre- Chemotherapy vs. Second Follow-Up	First Follow-Up vs. Second Follow-Up
TC (mg/dL)	Normal	14 (28.0)	12 (24.0)	11 (22.0)	0.564	0.405	0.796
	Abnormal	36 (72.0)	38 (76.0)	39 (78.0)			
TGs (mg/dL)	Normal	35 (70.0)	29 (58.0)	34 (68.0)	0.109	0.796	0.197
	Abnormal	15 (30.0)	21 (42.0)	16 (32.0)			
HDL-C (mg/dL)	Normal	36 (72.0)	35 (70.0)	41 (82.0)	0.705	0.096	0.058
	Abnormal	14 (28.0)	15 (30.0)	9 (18.0)			
LDL-C (mg/dL)	Normal	13 (26.0)	16 (32.0)	11 (22.0)	0.405	0.564	0.225
	Abnormal	37 (74.0)	34 (68.0)	39 (78.0)	0.405	0.564	0.225

**Table 4:** Correlations between BMI Index and lipids.

		BMI
Pre-chemotherapy TC	r	0.224
	*p*	0.118
Pre-chemotherapy TGs	r	−0.051
	*p*	0.727
Pre-chemotherapy HDL-C	r	−0.097
	*p*	0.501
Pre-chemotherapy LDL-C	r	0.294 *
	*p*	0.038
First follow-up TC	r	0.330 *
	*p*	0.019
First follow-up TGs	r	0.122
	*p*	0.397
First follow-up HDL-C	r	−0.219
	*p*	0.127
First follow-up LDL-C	r	0.376 **
	*p*	0.007
Second follow-up TC	r	0.543 **
	*p*	<0.001
Second follow-up TGs	r	0.112
	*p*	0.438
Second follow-up HDL-C	r	−0.2
	*p*	0.165
Second follow-up LDL-C	r	0.499 **
	*p*	<0.001

** Correlation is significant at the 0.01 level (2-tailed). * Correlation is significant at the 0.05 level (2-tailed).

## Data Availability

Not applicable.

## Public Involvement Statement

There was no public involvement in any aspect of this research.

## Guidelines and Standards Statement

This manuscript was drafted against the CONSORT guidelines for original clinical research. A complete list of reporting guidelines can be accessed via the equator network: https://www.equator-network.org/.
